# Effects of real-time feedback during decline walking on kinematic and kinetic gait parameters in a healthy population: study protocol for a randomized trial — up and down

**DOI:** 10.1186/s13063-021-05422-2

**Published:** 2021-07-22

**Authors:** Klaus Widhalm, Sebastian Durstberger, Peter Putz

**Affiliations:** FH Campus Wien, Health Sciences, Favoritenstrasse 226, 1100 Vienna, Austria

**Keywords:** Real-time feedback, Decline walking, Leg alignment, Kinematic, Kinetic, Treadmill

## Abstract

**Background:**

The control of the dynamic functional leg alignment (dFLA) and biomechanical load are important joint-related aspects regarding the development of osteoarthritis (OA). Research on level walking with feedback on load-related parameters has provided innovative treatment possibilities. With regard to walking on sloped surfaces, fundamental biomechanical knowledge exists. However, comprehensive data on the agreement of kinematics and kinetics of self-paced ramp versus sloped treadmill walking is lacking. Further, deeper insights into the control of the dFLA during decline walking and the usefulness of real-time feedback are missing.

**Methods/design:**

Thirty healthy participants aged between 18 and 35 years will be included. They will complete a three-dimensional gait analysis walking self-paced up and down on a 5-m ramp with a 10° inclination. Subsequently, speed-matched to ramp-up walking and self-paced 10° incline split-belt treadmill walking will be assessed. Afterwards, the participants will be observed under four different conditions of 10° declined walking on the same treadmill (a) self-paced walking, (b) self-paced walking with an internal focus of attention, (c) self-paced walking with real-time feedback, and (d) condition c speed-matched walking. The primary outcome parameter will be the frontal knee range of motion (fKROM). Secondary outcomes include the ground reaction force loading rate, spatial-temporal parameters, as well as sagittal, frontal and transversal kinematics, and kinetics for the lower extremities.

**Discussion:**

The findings aim at improving the understanding of the effects of real-time feedback on the control of the dFLA and lower limb loading. Following clinical practicable methods for effective feedback devices can be developed and evaluated. Additionally, the first dataset comparing kinematic and kinetic parameters for decline and incline ramp walking versus walking on an instrumented treadmill will be available for appropriate intervention planning.

**Trial registration:**

ClinicalTrials.govNCT04763850. Prospectively registered on 21 February 2021.

## Background

The prevalence of symptomatic osteoarthritis (OA) is estimated to be about 3–9% in European adults aged over 19 and increases towards 9–15% in those over 60 years [1]. Biomechanical loading has been identified as one of the highly relevant parameters concerning the development of knee OA and the management of conservative OA therapy [2, 3]. In this context, kinematic and kinetic parameters have been studied thoroughly during level walking. Few studies reported altered gait parameters for patients with OA in incline walking [4] and differences in kinetic parameters for increasing inclinations in healthy individuals [5]. However, declined walking is, due to higher vertical ground reaction forces (vGRF), even more challenging for the musculoskeletal system [6–8]. Specifically, knee and hip joint compression forces increase with the grade of declination [9]. The medio-lateral load distribution and loading velocity are generally associated with knee valgus/varus thrust [10], which was reported for level walking (LW) up to 6° in healthy persons [11, 12]. As during decline walking higher decelerating forces occur, an increase of valgus thrust might be induced. Following this, an inappropriate control of the dynamic functional leg alignment (dFLA) during decline walking would result in an increased frontal knee range of motion (fKROM) leading to inadequate shifted joint compression forces. These consequences are to be stressed, as the lateral knee compartment is more exposed to local pressure at initial contact than the medial one [13].

Besides one X-ray-based study, data for declined walking is missing in the current literature [10]. As loading and neuromuscular control have been shown to be important in the prevention and treatment of knee OA [14, 15], feedback is an effective tool supporting patients [16, 17]. Recently, it has been shown that feedback on loading rate is effective; a moderate retention effect could be observed as well [16]. Real-time feedback specifically targeting the sensorimotor control of the dFLA during walking has not been investigated yet.

Assessment of uphill or downhill locomotion has mainly been studied using instrumented ramps [6, 18]. Only a few studies have been conducted on instrumented treadmills with inclination [4, 19, 20], which display insufficiently comparable kinetic measurement technology. Furthermore, knowledge on the agreement of ramp and treadmill-derived kinetic and energy data is missing.

To our knowledge, the influence of motor control interventions as the internal focus of attention and real-time feedback on the aforementioned biomechanical factors in declined walking has not been studied before.

### Objectives

This randomized four-period crossover study aims at enhancing the understanding of the effects of real-time feedback on lower limb gait kinematics and kinetics. Furthermore, the agreement of kinematic and kinetic parameters of 10° decline and incline walking between ramp and treadmill will be assessed.

The primary research question is whether real-time feedback alters the frontal knee range of motion when compared to self-paced walking on the one hand and matched speed walking on the other hand. The secondary aspect focuses on the effect of lower limb loading in decline walking. Thirdly, parameters of interest will be compared between declined/inclined ramp and declined/inclined treadmill walking. Consequently, underlying hypotheses were phrased as follows:

#### Primary null hypothesis

Means of the outcome “frontal knee range of motion” are during treadmill decline walking equal across the four conditions (a) self-paced walking, (b) self-paced walking with an internal focus of attention, (c) self-paced walking with real-time feedback, and (d) condition c speed-matched walking. Predefined contrasts hypothesize that:

1. The outcome of self-paced walking with real-time feedback is equal to the speed matched walking (c=d)
2. The outcome of self-paced walking with an internal focus of attention and self-paced walking with real-time feedback is equal to self-paced walking (bc=a)
3. The outcome of self-paced walking with real-time feedback is equal to self-paced walking with an internal focus of attention (c=b)

#### Primary alternative hypothesis

Means of the outcome “frontal knee range of motion” are during treadmill decline walking NOT equal across the four conditions (a) self-paced walking, (b) self-paced walking with an internal focus of attention, (c) self-paced walking with real-time feedback, and (d) condition c speed-matched walking. Two-sided testing will be applied, as knowledge from the existing literature is limited. Predefined contrasts hypothesize that:

1. The outcome of self-paced walking with real-time feedback is NOT equal to speed-matched walking (c≠d)
2. The outcome of self-paced walking with an internal focus of attention and self-paced walking with real-time feedback is NOT equal to self-paced walking (bc≠a)
3. The outcome of self-paced walking with real-time feedback is NOT equal to self-paced walking with an internal focus of attention (c≠b)

#### Secondary null hypothesis

The means of the outcome “ground reaction force loading rate” are during treadmill decline walking equal across the four conditions (a) self-paced walking, (b) self-paced walking with an internal focus of attention, (c) self-paced walking with real-time feedback, and (d) condition c speed-matched walking. For this outcome, predefined contrasts are concordant with those of the primary hypothesis.

#### Secondary alternative hypothesis

The means of the outcome “ground reaction force loading rate” are during treadmill decline walking NOT equal across the four conditions (a) self-paced walking, (b) self-paced walking with an internal focus of attention, (c) self-paced walking with real-time feedback, and (d) condition c speed-matched walking. Two-sided testing will be applied, as knowledge from the existing literature is limited. For this outcome, predefined contrasts are concordant with those of the primary hypothesis.

Tertiary null hypothesis:

1. The agreement of continuous kinematic and kinetic outcomes of the lower limb (see list of outcome parameters) between decline ramp walking and speed-matched decline treadmill walking is lower than 0.75 (ICC).
2. The agreement of continuous kinematic and kinetic outcomes of the lower limb (see list of outcome parameters) between decline ramp walking and self-paced decline treadmill walking is lower than 0.75 (ICC).
3. The agreement of continuous kinematic and kinetic outcomes of the lower limb (see list of outcome parameters) between incline ramp walking and speed-matched incline treadmill walking is lower than 0.75 (ICC).
4. The agreement of continuous kinematic and kinetic outcomes of the lower limb (see list of outcome parameters) between incline ramp walking and self-paced incline treadmill walking is lower than 0.75 (ICC).

Tertiary alternative hypothesis:

1. The agreement of continuous kinematic and kinetic outcomes of the lower limb (see list of outcome parameters) between decline ramp walking and speed-matched decline treadmill walking is at least 0.75 (ICC).
2. The agreement of continuous kinematic and kinetic outcomes of the lower limb (see list of outcome parameters) between decline ramp walking and self-paced decline treadmill walking is at least 0.75 (ICC).
3. The agreement of continuous kinematic and kinetic outcomes of the lower limb (see list of outcome parameters) between incline ramp walking and speed-matched incline treadmill walking is at least 0.75 (ICC).
4. The agreement of continuous kinematic and kinetic outcomes of the lower limb (see list of outcome parameters) between incline ramp walking and self-paced incline treadmill walking is lower at least 0.75 (ICC).

## Methods/design

### Trial design

**Fig. 1:**
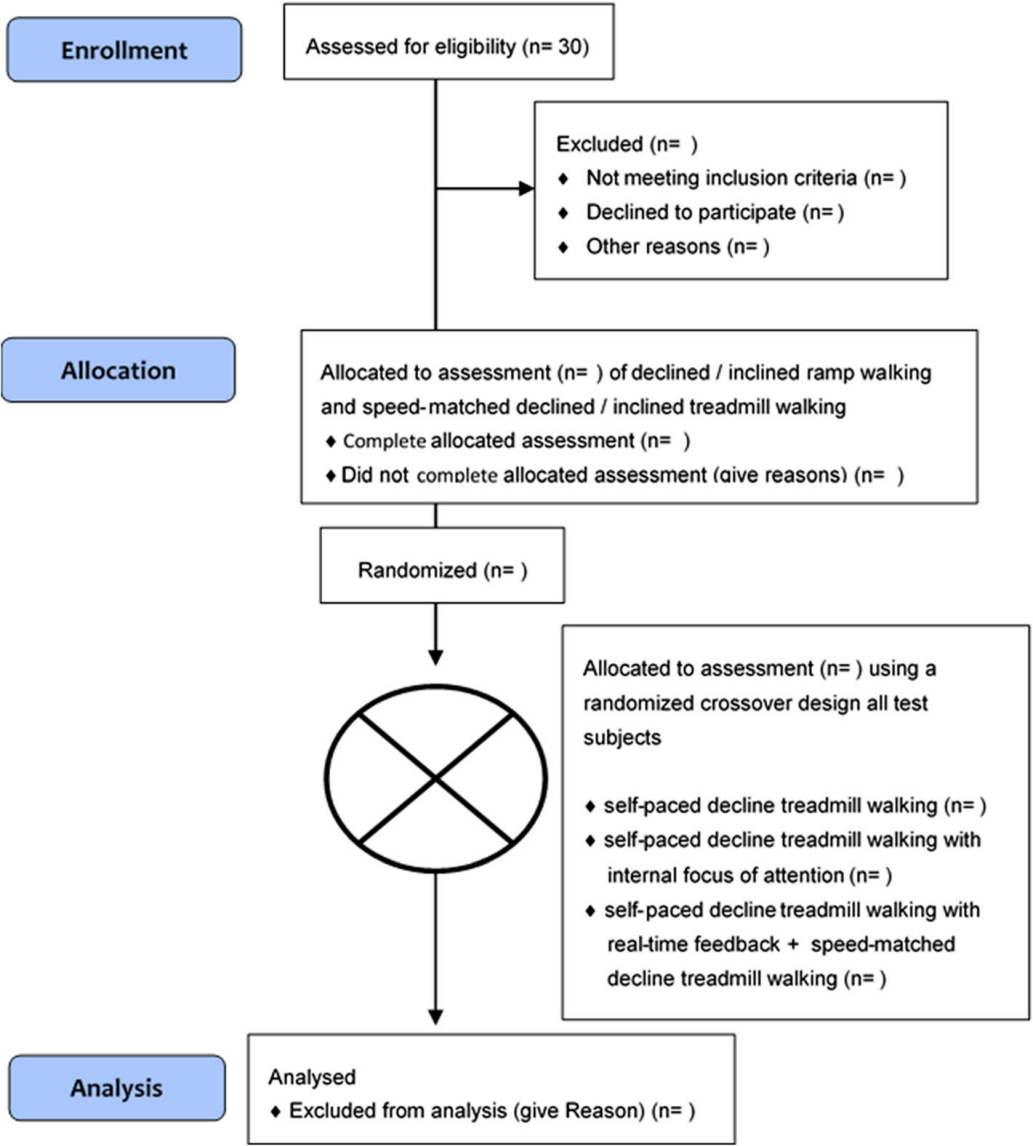
Flow diagram of the progress through the phases of the trial [22]

The study is conducted in the movement laboratory of the FH Campus Wien – University of Applied Sciences (Vienna, Austria). It is designed as a randomized four-period crossover study. Chronologically, the assessments will start with the tertiary hypotheses, examining agreements between declined/inclined ramp and treadmill walking. Thereafter, three out of four conditions (self-paced walking, self-paced walking with internal focus of attention, and self-paced walking with real-time feedback) are tested under block randomization of their six possible permutations (ABC, BCA, CAB, CBA, ACB, BAC). With three conditions (*n* = 3), this represents the smallest number of permutations, equaling a Williams design, where each condition precedes each other condition equally often and each treatment occurs equally frequently at each position [21]. The fourth corresponding condition (speed-matched walking) can technically not take place before the corresponding real-time feedback condition and is therefore exempted from the sequence randomization and always follows self-paced walking with real-time feedback. The numbers of participants assessed for eligibility, allocated to assessments, and analyzed will be recorded by a modified CONSORT flow chart [22], without consideration of losses to follow-up, due to the single visit study design. Figure 1 illustrates a flowchart of the trial design.

### Study population

As knowledge and studies concerning the effect of real-time feedback during decline walking are absent in the current literature, sample size calculation based on proven effects is not applicable. Therefore, the sample size was arbitrarily estimated at 25 participants, based on the number of participants included in topic-related studies, ranging from 14 to 40 [16, 23–25]. Considering a dropout rate of 15%, the recruitment of 30 participants was scheduled. Participants may not be able to complete the assessment battery, e.g., due to blistering of the heels. In addition, a lack of sufficient force plate strikes may disable data processing. Healthy participants will be included, according to the following inclusion criteria:

1. Age from 18 to 35 years
2. Body mass index from 18.5 to 29.99 kg/m^2^
3. No chronic joint disease(s) and/or OA surgery
4. No neuro-motor disease(s).

Participants will be excluded from randomization and the following assessments in case of the following:

1. Limited sagittal range of motion at the ankle, knee, or hip joint
2. Limited hamstring flexibility
3. Increased asymmetry concerning the aforementioned range of motion or flexibility, which could possibly affect the physiological level or ramp walking.

Participants will be excluded from the analysis in case of the following:

1. Non-physiological and non-symmetrical gait patterns
2. Severe outliers (more than two standard deviations) concerning main outcome parameters.

### Recruitment, informed consent, and randomization

Students of the bachelor course physiotherapy of the FH Campus Wien, University of Applied Sciences, will be addressed with the study information via in-house corridor monitors and an email newsletter. If interested, they may contact the primary investigator. A research team member will check eligibility and provide study details. An assessment date will be agreed upon in case of willingness and eligibility of the potential participant. Written informed consent will be obtained from the participants prior to the assessments. Subsequently, the principal investigator will check for possible physical limitations following a structured interview protocol concerning the musculoskeletal health status; the functional flexibility status of the lower limb will be assessed as well. The participants will randomly be assigned to one of the six sequences, by means of a prepared sequence list as derived from a random sequence generator online tool [26]. Due to the nature of the studied conditions, it will not be possible to blind participants. No measures are foreseen regarding allocation concealment or blinding of researchers. A participant will pass through all assessments within 1 day. Shopping vouchers of € 30.00 will be offered as an incentive for participation.

### Ethical and data protection aspects

With respect to participating students, a teacher–student relationship might exist between researchers and participants. Besides adherence to general ethical principles, i.e., voluntary participation, data protection, and confidentiality, provision of contact details of the research team, it will be emphasized in the course of obtaining informed consent, that refusing or withdrawing consent will not have any consequences on the remaining academic education. Participants will be assigned a continuous study code (UD01 – UD30) to protect identifiable data. The assignment key will be locked in the study manager’s office, separate from identifiable study data.

### Safety considerations and participant insurance

No adverse events are to be expected. The principal investigator will instruct the participants on treadmill walking and the safety system. The latter will consist of a safety harness that will be attached to a suspension system, two handrails, light gates at the front and back of the belt, and an emergency button at the operating desk. The participants will be granted 5 min of familiarization with the treadmill, which is operated with matched walking speed from the ramp walking trials. A clinical trial insurance will be taken out for all participants.

Having finished the active phase of the study, participants’ skin will be viewed after removing skin markers and the safety-harness. Further, they will be asked about their well-being.

Due to the SARS-2 pandemic and the current epidemiological situation, examiners and participants will have to wear an FFP2 mask; participants will be asked concerning their health status, and examiners will use a SARS-2 antigen quick check on each assessment day.

### Data collection and intervention

**Fig. 2:**
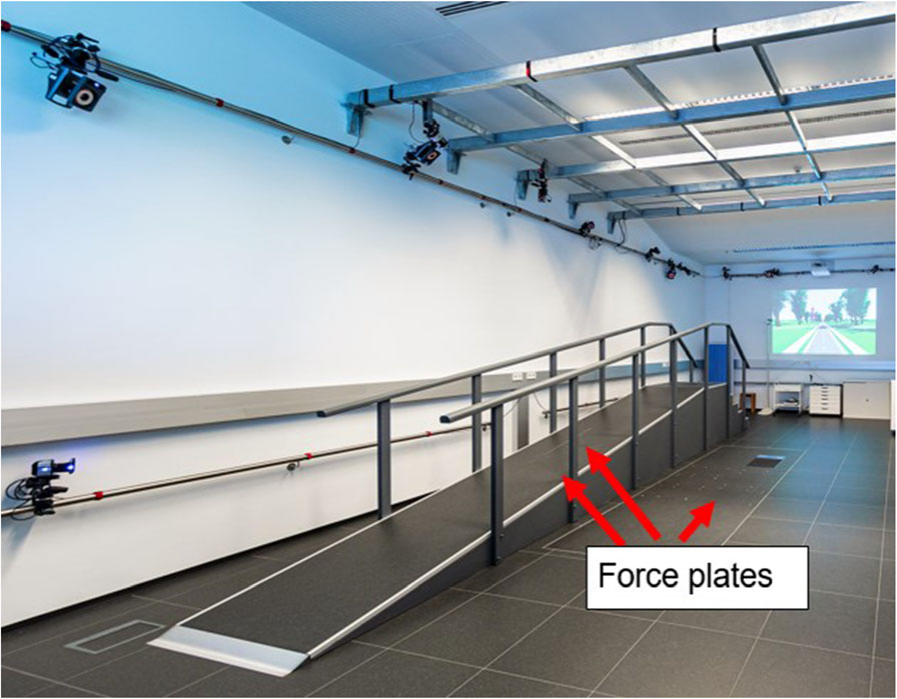
Laboratory setting for ramp and level walking

At first, the height will be measured with a stadiometer to the nearest 0.5 cm (SECA 213), and the weight with a medical scale (Marsden M-420). For reasons of biomechanical modeling, inter-anterior superior iliac spine distance will be taken using a spreading caliper with rounded ends (GPM 0–300 mm). Furthermore, the lower limb joint range of motion in the sagittal plane and functional hamstring flexibility will be assessed. Thereafter, all participants will be equipped with the Upper Body Plug-In-Gait Marker Set and the Modified Cleveland Clinical Marker Set. Thus, a total of 43 retroreflective markers on bony landmarks and 16 markers on four rigid clusters on thighs and shanks will be applied. Five level walking trials will be captured to evaluate whether the gait pattern is within a physiological range. Participants will have to walk on a 5-m ramp with 10° inclination (Fig. 2), which is equipped with 24 infrared cameras (Vicon, Oxford, UK) and two force plates (Kistler, Winterthur, Switzerland).

**Fig. 3:**
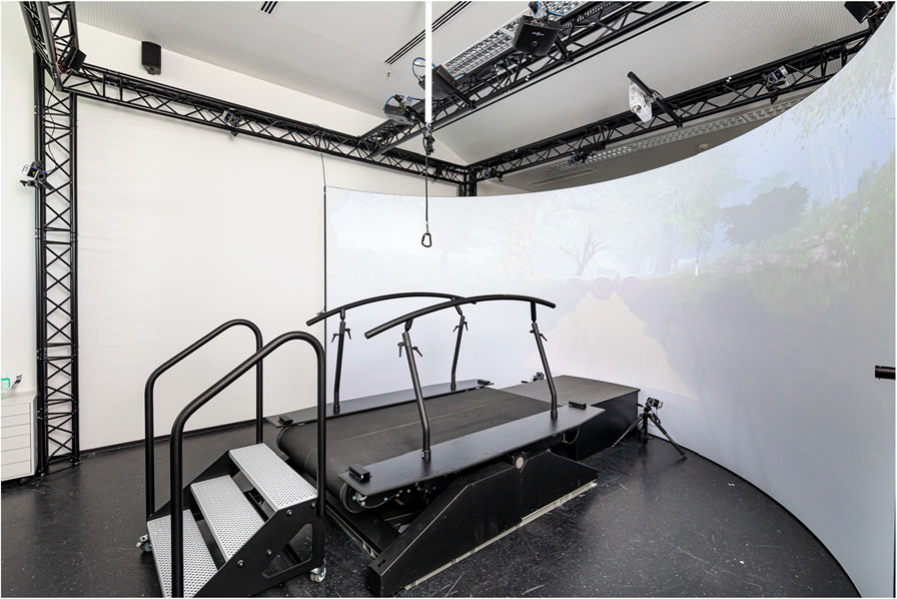
GRAIL system illustration

After seven left and seven right foot strikes on the force plates for up and down walking each, the participants will be guided to the Gait Real-time Analysis Interactive Lab (GRAIL, Motekforce Link, Amsterdam, Netherlands), which is located next door. The GRAIL is a dual-belt treadmill equipped with two force plates, a 180° virtual reality screen and a motion capturing system with ten infrared cameras (Fig. 3).

After the familiarization phase on the treadmill with an inclination of 10° and with matched speed of inclined ramp walking, three times a period of 10 s will be recorded within 3 min. The investigator will explain the self-paced mode of the treadmill to the participant afterwards. The self-paced mode will adapt the belt speed automatically based on the position of the participant on the treadmill. Following a familiarization phase, data will be recorded as before.

This procedure will be repeated on the treadmill with a declination of 10° speed matched with decline ramp walking. Thereafter, the three following tasks will be performed in the order as specified in the sequence list.

#### Self-paced decline walking

The participants will be instructed to walk on the declined treadmill with self-selected speed. They will complete a 5-min familiarization period and a 3-min measurement period. Within the measurement phases, three times a period of 10 s of self-paced walking will be recorded.

#### Self-paced decline walking with an internal focus of attention

The participants will be instructed to walk on the declined treadmill with self-selected speed. During walking, they will have to concentrate on an internal focus of attention, such as keeping their spine as long towards the ceiling as possible. The participants will complete a 5-min familiarization period and a 3-min measurement period. In the measurement phase, three times a period of 10 s of self-paced walking will be recorded.

#### Self-paced decline walking with real-time feedback, followed by speed-matched decline walking

**Fig. 4:**
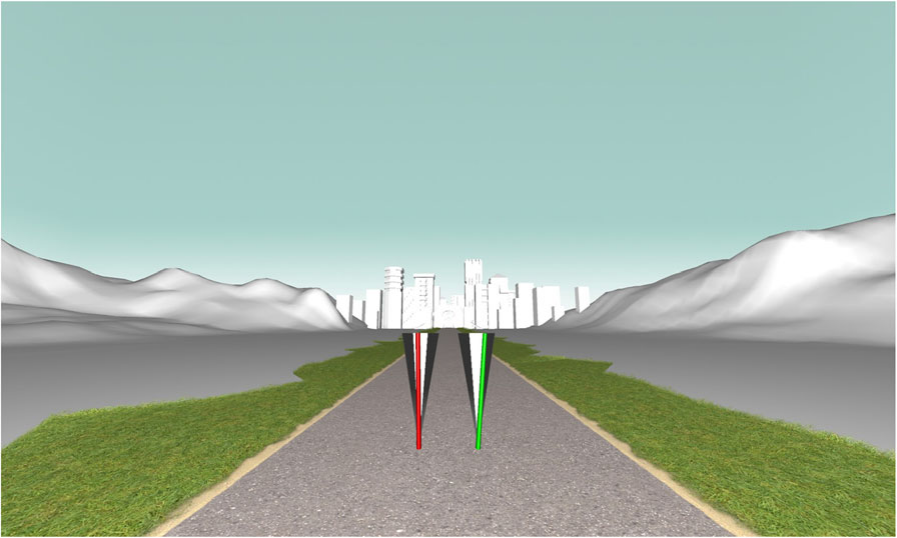
Visualization of the presented feedback

The participants will be instructed to walk on the declined treadmill with self-selected speed. During walking, they will have to concentrate on specific real-time feedback presented on the projection screen. This feedback visualization will represent the actual simplified frontal knee alignment movement for the loading-response phase. The feedback parameter is calculated as an angle between projections of two vectors on the plane, normal to the gait direction. The origin of both vectors will be the marker at the lateral femoral condyle. One vector will point to the bottom marker on the thigh cluster and the other vector to the top marker on the shank cluster. To avoid an offset due to the cluster placement, the feedback value will be set to zero in participants’ individual neutral stand position. Participants are instructed to keep the stick steady within the circular segment when loading their legs (Fig. 4). By keeping the stick, which represents the frontal knee alignment, steady, an increased control of possible knee valgus thrust is intended. The participants will complete a 5-min familiarization period and a 3-min measurement period. In the measurement phases, three times a period of 10 s of self-paced walking will be recorded. After this task, a controlled trial will take place with matched speed and without any feedback instruction.

During the intervention phase, the participants will be observed for any changes concerning their movement behavior and regularly will be asked about their well-being. If any sign of harm (i.e., blistering at the heel) or movement behavior change (i.e., arbitrary altering their gait pattern) would occur, the principal investigator will ask the participant on this and decide on a possible discontinuing of the intervention according to the eligibility criteria. In case of discomfort at the heel, the application of a blister plaster will be possible. Such unaccepted occurrences will be noted in the case report form for reasons of data quality check in the analyzing phase. If participants discontinue during the intervention or deviate from the intervention protocol, recorded data has to be excluded from the analysis. As walking on the above-described instrumentation has been tested intensively before, the expected drop-out rate should cover a possible loss of participants.

### Outcomes

Height, bodyweight, and inter-anterior superior iliac spine distance will be measured at the beginning of all sessions. Furthermore, the flexibility of hamstrings and triceps surae will be assessed clinically. Gait analysis outcome parameters will be extracted from the biomechanical model, which will be generated from the marker positions. The hip joint center position will be predicted according to Davis [27]. Knee and ankle joint center positions will be defined as the midpoint between lateral and medial knee or ankle markers, and the vector between those two markers will define the knee and ankle axis. The primary outcome of this study is the frontal knee range of motion during the stance phase. The stance phase will be defined between heel-strike and toe-off of the observed leg. A second focus is on the ground reaction force-loading ratio. Thirdly, the kinematics for the sagittal, frontal, and transversal plane for the hip, knee, and ankle and the external moments for the sagittal and frontal plane for the hip, knee, and ankle will be assessed as well as step time, step length, step width, cadence, and walking speed (Table 1).

**Table 1:** List of tertiary outcome parameters

Area	Outcome measure
**Kinematic**	Sagittal, frontal, and transversal hip angle range of motion (°)
	Minimum sagittal hip angle (°)
	Maximum sagittal hip angle (°)
	Sagittal, frontal, and transversal knee angle range of motion (°)
	Minimum sagittal knee angle (°)
	Maximum sagittal knee angle (°)
	Sagittal ankle angle range of motion (°)
	Minimum sagittal ankle angle (°)
	Maximum sagittal ankle angle (°)
	Sagittal, frontal, and pelvis angle range of motion (°)
	Sagittal, frontal, and trunk angle range of motion (°)
	Minimum sagittal trunk angle (°)
	Minimum sagittal trunk angle (°)
**Kinetics**	First peak load (N)
	Time point of first peak load (% stance phase)
	Average loading rate (N s^−1^)
	Maximum loading rate (N s^−1^)
	Knee joint forces (N/kg)
	Minimum sagittal, frontal, and transversal hip external moments (Nm/kg)
	Maximum sagittal, frontal, and transversal hip external moments (Nm/kg)
	Minimum sagittal, frontal, and transversal knee external moments (Nm/kg)
	Maximum sagittal, frontal, and transversal knee external moments (Nm/kg)
	Minimum of sagittal ankle external moment (Nm/kg)
	Maximum of sagittal ankle external moment (Nm/kg)
	Frontal knee abduction moment impulse (Nm/kg s)
	Frontal knee adduction moment impulse (Nm/kg s)
**Spatio-temporal parameters**	Walking velocity (m s^−1^)
	Cadence (steps min^−1^)
	Step length (m)
	Step width (m)
	Stance phase duration (% gait cycle)
	Swing phase duration (% gait cycle)

Kinematic data will be time normalized from heel strike to heel strike, and kinetic data from heel strike to toe-off. Individual participant data will be expressed as average per condition. Time normalization, data processing, and parameter calculations will be done with self-developed scripts in MATLAB 2018a (The Mathworks, Natick, MA, USA). This MATLAB script includes steps for data quality checks, which are additionally performed by the four-eye principle. According to the study design and the type of intervention, there is no need for an independent data monitoring committee and monitored interim analyses.

### Statistical analysis

All metric parameters will be analyzed using SPSS in its recent version (IBM Corporation, Armonk, NY, USA). Data will be tested for normality using the Shapiro-Wilk Test, and graphical inspections of Q-Q plots will be performed. Regarding the primary and secondary hypotheses, differences between the four conditions (decline walking, internal focus of attention, real-time feedback, and matched speed walking) will be tested using a one-factorial analysis of variance with repeated measures (rmANOVA). The assumption of sphericity will be tested with Mauchly’s test. The Greenhouse-Geisser correction will be applied in case of a violation of the sphericity assumption. Contrasts are used to test the pre-specified hypotheses as stated under “Objectives” above. For the overall rmANOVA, ω^2^ will be calculated, and r as the effect size measure of the contrasts. One-dimensional statistical parameter mapping (1dSPM) will be used to examine the continuous parameters of the gait analysis [28]. Regarding the tertiary hypothesis, the differences between ramp-walking and matched speed/self-paced walking on the inclined/declined treadmill will be analyzed using paired two-tailed t tests, and agreement will be tested using (intraclass correlation) ICC [3.1, absolute agreement, single measures] with standard error of measurement (SEM) of the ICC. Within the tertiary hypothesis, there are four paired t tests and four ICCs per outcome, as summarized in Table 1. Additionally, Bland-Altman plots with 95% limits of the agreement will be created for selected parameters (three planes of knee range of motion and spatio-temporal parameters as stated in Table 1). All statistical tests other than those related to the primary and secondary hypotheses are considered exploratory, and statistical testing will be limited to tests as pre-specified by the hypotheses. The Bonferroni-Holm method is used with regard to the correction for multiple testing. Tests other than those related to the primary and secondary hypotheses are considered exploratory and are consequently exempted from the multiple testing correction. To facilitate the parametric analysis in a homogeneous sample, participants will be excluded from the analysis in case of non-physiological and non-symmetrical gait patterns, and severe outliers (more than two standard deviations) concerning main outcome parameters. Two-tailed testing is applied where applicable. Alpha will be set to 0.05; however, exact *p* values will be reported.

## Discussion

This study will provide novel knowledge on the effect of real-time feedback during declined walking on the control of the dFLA using the visible parameter frontal knee range of motion. Using the fKROM parameter, an increase in anticipatory muscle activation prior to initial contact and loading of the following stance limb can be expected. Consequently, the hip adduction and internal rotation-induced valgus thrust during the loading-response phase should decrease, implying a stabilized dFLA. In contrast, similar studies have been conducted for level walking using the external moments as feedback parameters, which sometimes resulted in altering gait parameters like lateral trunk lean or step width.

Furthermore, a possible difference in the effectiveness of the presented feedback on the dFLA compared to an internal focus of attention instruction will be studied. Particularly in a population trained in the internal focus of attention for optimizing motor control, direct feedback on the dFLA might be inferior. The results may inform the design of consecutive studies on specific target populations.

The authors anticipate the following study limitations. The condition of speed-matched walking cannot be included in the randomization procedure. Due to the nature of the studied conditions, blinding participants will not be possible. Another limitation is that different force plates are used for ramp walking and treadmill walking. Besides, the continuous quality of declined treadmill force plate data cannot be guaranteed. Thereby, a small systematic error might affect the kinetic outcome. The findings of this study will apply only to healthy young adults. As participants will be physiotherapy students, who are expected to present a higher motor control level compared to matched controls, there could be a bias for adapting to the applied form of feedback during decline walking.

Focusing on the tertiary hypothesis, the present study will be the first one providing data on the agreement of kinematic and kinetic parameters for decline and incline walking on a ramp compared to an instrumented treadmill. This will help in clinical and research decision-making on which assessment is appropriate.

### Trial status

The ethics committee of the Medical University Vienna has approved the study protocol. Recruitment of participants started on 26 January 2021 and will be open until 30 participants have begun the intervention phase. Data collection and intervention are scheduled to take place between the end of February and the end of May 2021. Each participant has a 2-h active phase from giving informed consent until the end of the intervention.

## Data Availability

A manuscript will be submitted to a journal with a peer review process aiming for an open-access publication. Coded raw data will be made available upon reasonable request and for non-commercial purposes after the publication of the results.

## Abbreviations

dFLA: dynamic Functional Leg Alignment
fKROM: frontal Knee Range of Motion
GRAIL: Gait Real-time Analysis Laboratory
ICC: Intraclass correlation coefficient
LW: Level walking
OA: Osteoarthritis
SEM: Standard error of measurement
vGRF: vertical Ground Reaction Force
rmANOVA: One-factorial analysis of variance with repeated measures
1dSPM: One-dimensional statistical parameter mapping
KW: Klaus Widhalm
SD: Sebastian Durstberger
PP: Peter Putz
